# GNG4 Promotes Tumor Progression in Colorectal Cancer

**DOI:** 10.1155/2021/9931984

**Published:** 2021-10-15

**Authors:** Liang Liang, Jun Huang, Ming Yao, Lei Li, Xiao-Jian Jin, Xiao-Yong Cai

**Affiliations:** Departments of General Surgery, The Second Affiliated Hospital of Guangxi Medical University, Nanning 530007, Guangxi Zhuang Autonomous Region, China

## Abstract

Colorectal cancer is a common digestive system tumor, which lacks effective therapeutic targets and biomarkers to accurately determine the prognosis. Sequencing data, immunohistochemistry, and Kaplan–Meier analysis were used to explore GNG4 clinical significance in colorectal cancer. And, through in vitro experiments, the effects of GNG4 on cell behaviors were investigated. The results showed that the mRNA and protein expression levels of GNG4 in patients with colorectal cancer were significantly higher than in normal people. The patients with high GNG4 expression had a worse prognosis than patients with low GNG4 expression. The in vitro experiments presented that after downregulating the expression of GNG4, proliferation, migration, and invasion of SW-620 colon cancer cells were all significantly reduced, apoptosis was significantly increased, and the cell cycle was blocked in the S phase. In summary, GNG4 may be of importance in the therapy of the colorectal cancer; therefore, targeting GNG4 may have certain clinical value in the treatment of colorectal cancer.

## 1. Introduction

Colorectal cancer is a major neoplasm of the digestive tract, which has a high morbidity and mortality rate in China [1]. In recent years, with the change in environment and living habits, the incidence of colorectal cancer has seen a significant upward trend. Although great progress has been made in colon cancer treatments, the survival of colon cancer patients is not satisfactory [2]. Therefore, elucidating pathogenesis of colorectal cancer to find new therapeutic targets is particularly important for colorectal cancer survival.

The guanine nucleotide-binding protein (G protein) is a regulator of transmembrane signaling pathways. G protein transduces information via multiple signal pathways, including MAPK, PI3K, and RhoGEF pathways [3]. The gamma chain of G protein, such as G protein subunit gamma 4 (GNG4), one of the fourteen *γ* subunits of human G protein, is necessary for GTPase activity, GTP substitution for GDP, and G protein-effector interaction. In recent years, many studies investigated the role of GNG4 in tumors. GNG4 is reported to be hypermethylated in bladder cancer and glioblastoma [3, 4], and its expression significantly decreased. Overexpression of GNG4 also significantly inhibits tumor proliferation [3]. However, studies have shown that the expression of GNG4 is significantly upregulated in colorectal cancer, although its role is unclear [5, 6].

In our study, we report that the expression of GNG4 is upregulated in colon cancer. The expression of GNG4 was negatively related to overall survival (OS) of patients with colon cancer. The results show that silencing the expression of GNG4 caused an increase in apoptosis of SW-620 cells, which may inhibit the cell proliferation. In addition, reducing the expression of GNG4 inhibited cell migration and invasion and blocked the cell cycle in the S phase. Thus, a deeper understanding of the molecular mechanism of GNG4 in the development of colorectal cancer may lead to new therapeutic strategies.

## 2. Materials and Methods

### 2.1. Collection of Clinical Samples

A total of 55 pairs of colorectal cancer and normal tissues were obtained from the Second Affiliated Hospital of Guangxi Medical University (Table 1). The tissues were fixed in formaldehyde and embedded in paraffin for preservation. Tissue specimens were stored at −80°C. The study was approved by the ethics committee, and all patients signed informed consent.

### 2.2. Expression of GNG4 in Colorectal Cancer Tissues

The GEPIA database (https://gepia.cancer.pku.cn/) was used to analyze the expression data of GNG4 in colon and rectal cancer [7]. GEPIA data were collected from TCGA and GTEx databases, including 275 colon cancer and 349 normal colon tissues, and 92 cases of rectal cancer and 318 cases of normal rectal tissues. In addition, the OS and disease-free survival (DFS) of colorectal cancer patients relative to GNG4 expression were predicted through GEPIA and median cases was set as group cutoff.

### 2.3. Quantitative RT-PCR

The total RNA isolation kit (RC101, Vazyme, China) was used to isolate total RNA. HiScript II QRT SuperMix (R223, Vazyme, China) was used for reverse transcription into cDNA. ChamQ Universal SYBR qPCR Master Mix (Q711, Vazyme, China) was used as SYBR fluorescent dyes. The whole process was detected by the ABI Prism 7500 rapid sequence detection system (Applied Biosystems, USA). The primers for GNG4 were 5′-ACCCACCGTGGAAGCTGAAG-3′ and 5′-CCCAAGCAAGGGTCCAGGTA-3′, and the primers for *GAPDH* were 5′-GCTCTCTGCTCCTCCTGTTC-3′ and 5′-CGACCAAATCCGTTGACTCC-3′. Each experiment was performed in triplicate. GNG4 expression was normalized to GAPDH expression in each sample.

### 2.4. Immunohistochemistry

Immunohistochemistry was performed in 55 pairs of colorectal cancer and normal tissues. Immunohistochemical detection was performed using the Universal two-step detection kit (PV-9000, Zsbio, China). Slices were treated with EDTA buffer for antigen retrieval at 100°C temperature for 5 min. Then, they were incubated with 3% H_2_O_2_ for 10 minutes at room temperature to inactivate endogenous peroxidase activity. The GNG4 antibody was diluted at 1 : 200 (bs-13245R, Bioss, China) and incubated for 12 h at 4°C. The reaction solution was added according to the instructions of the reagents, and finally, DAB was used as a chromogenic substrate. A negative control was also established using the same experimental conditions. Three fields with 200× magnification were randomly selected, and results were calculated by the immunoreactivity score [8].

### 2.5. Cell Culture and Transfection

The SW-620 (colon cancer cell) line was obtained from the cell bank of the Chinese Academy of Sciences, Shanghai, China. SW-620 cells were cultured in RPMI-1640 at 37°C in 5% CO_2_, supplemented with 10% fetal bovine serum (FBS). When the cells had grown to 80% area in six-well plates, they were transfected with lentiviral-based Lipofectamine 2000 (Invitrogen, Carlsbad) according to the manufacturer's protocol.

### 2.6. Cell Proliferation

The MTT (M5655, Sigma, US) assay was used to detect the effect of downregulation of GNG4 on cell viability. In brief, SW-620 cells were cultured in 96-well plates and incubated for 24 h. Then, SW-620 cells were transfected with shGNG4 or shRNA-NC, for 24 h, 48 h, and 72 h. MTT was added for incubation, and the absorbance was measured at 568 nm. The experiment was carried out in three sets of independent repetitions.

### 2.7. Apoptosis Assay

After 48 h of transfection with shGNG4 or shRNA-NC, SW-620 cells were trypsinized. To analyze apoptosis, we collected and rinsed the cells twice with PBS and then used the AnnexinV-APC/7-AAD cell apoptosis detection kit (KGA1026, KeyGen, China) for detection according to the instructions. Finally, the cell apoptosis was detected using a flow cytometer.

### 2.8. Cell Cycle Detectiom

After 72 hours of transfection of GNG4-shRNA, the cell cycle phase was evaluated by fluorescence activated cell sorting (FACS). First, the cells were treated with Triton X-100 and RNase, then the cell nuclei were stained with propidium iodide (PI), and finally, the DNA content was measured using a cell cycle kit (KGA512, KeyGen, China). At least 30,000 cells were analyzed in each experiment.

### 2.9. Migration and Invasion Assays

SW-620 cells were transfected with shRNA-GNG4 or shRNA-NC for 72 h. The polycarbonate membranes Transwell and Matrigel were used to simulate the environment of cell invasion. Resuspending the cells, we added 6 × 10^4^ cells to 200 *μ*l serum-free medium and spread them in the upper chamber. Then, 800 *μ*l of the complete medium containing FBS was added to the lower chamber. After 24 h of incubation, when the cells penetrated into the lower chamber, we used a cotton swab to gently wipe off the Matrigel and cells in the upper chamber. The membrane was fixed with alcohol and stained with 0.1% crystal violet. The Transwell chamber was observed and photographed with an upright microscope, and multiple fields of view were randomly selected for cell counting (200× magnification). The experiment was repeated three times independently. In addition, only the Transwell chamber was used for cell migration experiment.

### 2.10. Statistical Analysis

All statistical analyses were performed by SPSS software (SPSS 22.0, Chicago, IL). All data were expressed as means ± standard deviation (SD). Student's *t*-test and analysis of variance (ANOVA) were used to assess the differences in the corresponding groups. The difference was considered to be statistically significant when the *P* value was less than 0.05.

## 3. Results

### 3.1. GNG4 mRNA and Protein Expression Levels in Colorectal Cancer

**Figure 1:**
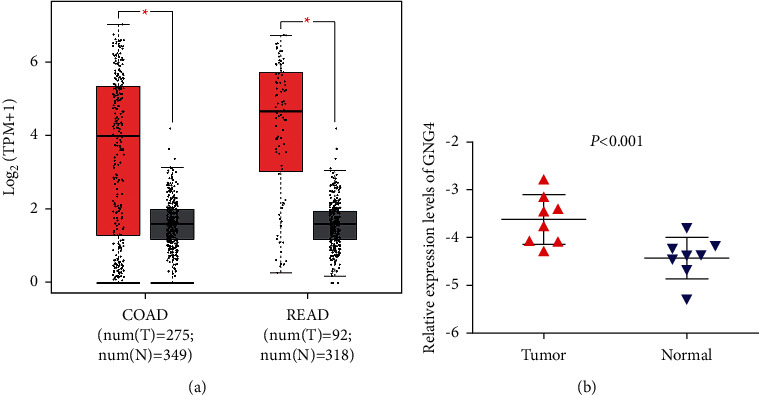
Expression of GNG4 in colorectal cancer. (a) GEPIA expression data of GNG4 in colorectal cancer (red) and normal adjacent tissues (gray). (b) The expression of GNG4 in colorectal cancer and adjacent normal tissues by RT-PCR. COAD: colon adenocarcinoma. READ: rectum adenocarcinoma.

**Figure 2:**
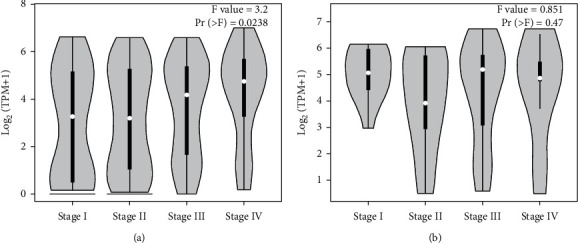
Pathological stage plot of GNG4 expression levels in COAD and READ. (a) The expression level of GNG4 according to the clinical stage of colon cancer (*P*=0.024). (b) There was no significant correlation between the expression level of GNG4 and the clinical stage of rectal cancer (*P*=0.470). Pathological stage plots were obtained from GEPIA, and differential gene expression was analyzed by one-way ANOVA (https://gepia.cancer-pku.cn/).

**Figure 3:**
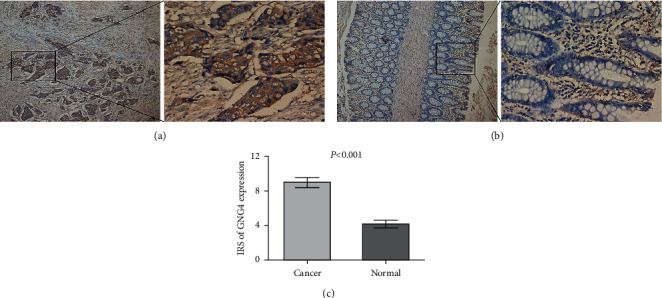
Protein expression levels of GNG4 in colorectal cancer. The immunohistochemical analysis of 55 pairs of colorectal cancer and normal adjacent tissues showed that the expression of GNG4 in colorectal cancer was significantly higher than that in adjacent normal tissues (*P* < 0.001). (a) Cancer tissue. (b) Normal tissue. (c) Immunoreactivity score (IRS) of GNG4 expression (magnification: 100× (left), 400× (right)).

GEPIA results showed that the expression of GNG4 in colon cancer was obviously higher than that in normal tissues; similarly, the expression of GNG4 in rectal cancer was also significantly increased (Figure 1(a)). Moreover, we verified the expression of GNG4 in colorectal cancer with RT-PCR. The results obtained were consistent with the sequencing results of GEPIA data (Figure 1(b)). The expression of GNG4 in colon cancer increased with the cancer stage (Figure 2(a)). However, the expression of GNG4 showed no significant difference in different stages of rectal cancer (Figure 2(b)). Finally, immunohistochemical analysis of 55 pairs of colorectal cancer and normal tissues found that the expression level of GNG4 in colorectal cancer was obviously higher than that in normal tissues (*P* < 0.001) (Figure 3(a)–3(c)).

### 3.2. Effect of GNG4 Expression on the Prognosis of Colorectal Cancer Patients

**Figure 4:**
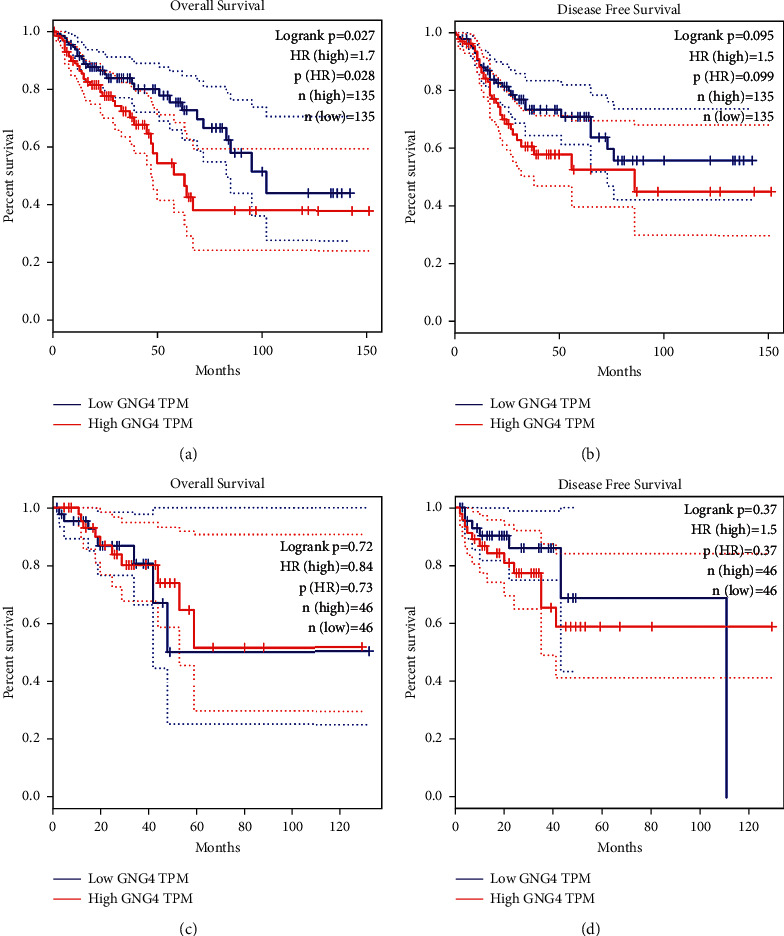
Kaplan–Meier analysis of overall survival (OS) and disease-free survival (DFS). Relationship between GNG4 mRNA expression level and (a) OS of colon cancer patients, (b) DFS of colon cancer patients, (c) OS of rectal cancer patients, and (d) DFS of rectal cancer patients.

According to the Kaplan–Meier plot with median expression of GNG4, the OS rate of colon cancer patients with high expression of GNG4 was lower than that of patients with low expression of GNG4 (*P*=0.027) (Figure 4(a)). However, there was no significant difference in disease-free survival time between colon cancer patients with high GNG4 expression and low GNG4 expression (Figure 4(b)). Moreover, there was no significant difference between high and low expression of GNG4 and the total survival time and DFS time of patients with rectal cancer (Figures 4(c) and 4(d)).

### 3.3. Effect of GNG4 on Cell Proliferation

**Figure 5:**
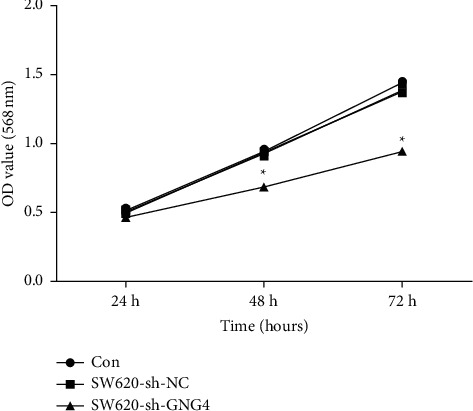
shRNA-mediated knockdown of GNG4 inhibited the proliferation of SW-620 cells by MTT assay; ^*∗*^*P* < 0.05.

After shGNG4 was transfected into SW-620 cells for 48 h, the growth activity of the cells was detected by MTT assay. The cell proliferation rate of shGNG4 cells after 48 h and 72 h was obviously lower than that of control cells (shRNA-NC, *P* < 0.05) (Figure 5). MTT assay results showed that silencing of the GNG4 gene inhibited the proliferation of colon cancer cells.

### 3.4. Effect of GNG4 on Migration and Invasion

**Figure 6:**
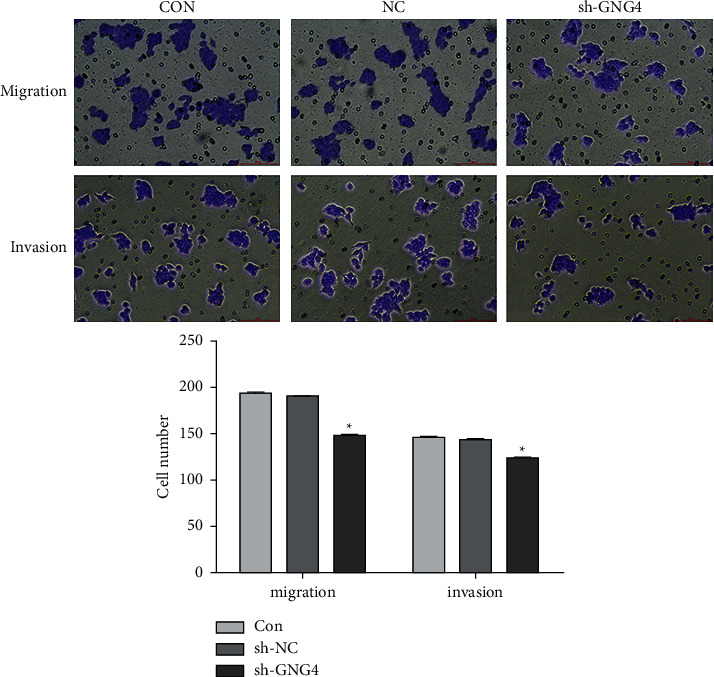
Effect of GNG4 on the migration and invasion of SW-620 cells. Compared with the control groups, knockdown of GNG4 expression significantly inhibited cell migration and invasion; ^*∗*^*P* < 0.05.

In order to explore the effect of GNG4 on the migration and invasion of SW-620 cells, Transwell assays were performed. Compared with the control group, the migration and invasion ability of shGNG4 cells were significantly reduced (*P* < 0.05) (Figure 6). This suggests that silencing of the GNG4 gene has a significant inhibitory effect on SW-620 cell migration and invasion.

### 3.5. Effect of GNG4 on Apoptosis

**Figure 7:**
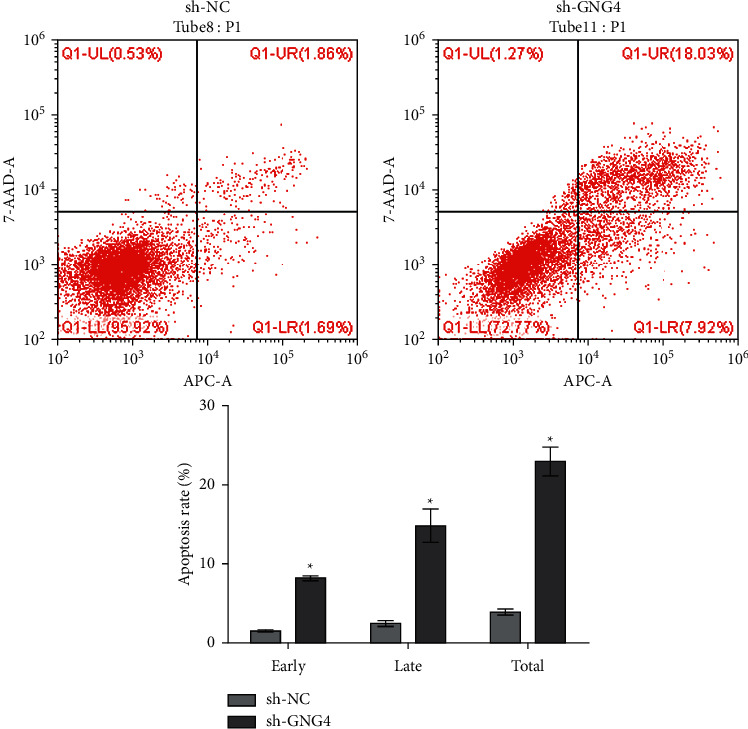
Effect of GNG4 expression on apoptosis of SW-620 cells. Downregulation of GNG4 significantly promoted the increase of cell apoptosis rate; ^*∗*^*P* < 0.05.

In order to explore the effect of GNG4 on cell apoptosis, cells transfected with shGNG4 for 48 h were collected for analysis. FITC-Annexin V staining showed that, compared with the control groups, silencing of GNG4 significantly increased both early and late apoptosis, as well as the total number of apoptotic cells (*P* < 0.05) (Figure 7).

### 3.6. Effect of GNG4 on the Cell Cycle

**Figure 8:**
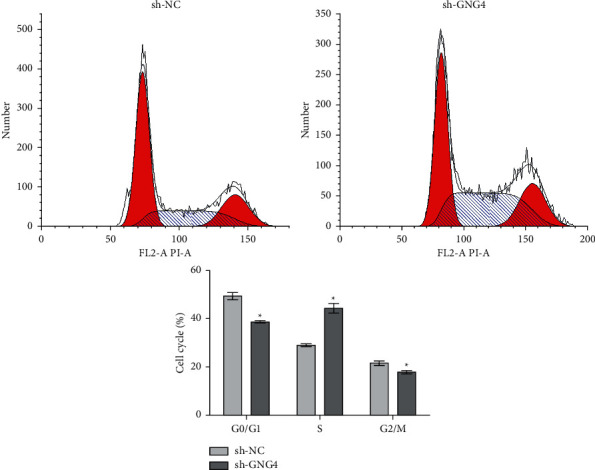
Effect of GNG4 on the cell cycle in SW-620 cells. Downregulation of GNG4 significantly decreased the ratio of G0/G1 and G2/M phase cells and increased the ratio of S phase cells; ^*∗*^*P* < 0.05.

Cell cycle assays were performed using SW-620 cells that had been transfected with shGNG4. Compared with the control group, the proportion of cells in the G0/G1 and G2/M phases was significantly reduced, while the ratio of cells in the S phase was significantly increased (Figure 8). This suggests that inhibiting the expression of GNG4 could induce cell cycle arrest of colon cancer cells in the S phase.

## 4. Discussion

**Figure 9:**
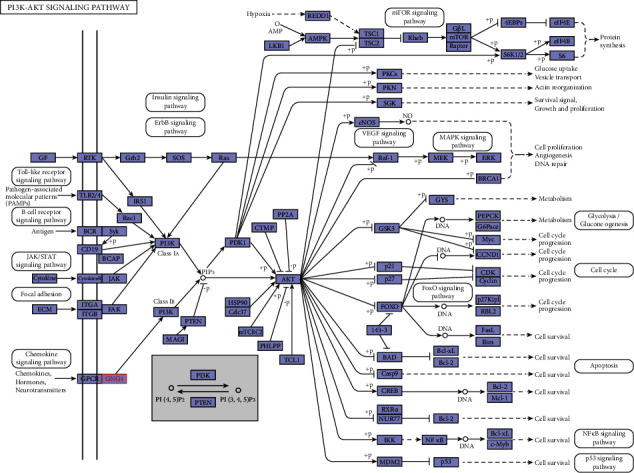
GNG4 may affect colorectal cancer progression through the PI3K/AKT signaling pathway. GNG4 is marked in red font.

Colorectal cancers are often malignant tumors with poor prognosis and frequent metastases. Radiotherapy and chemotherapy are also not especially helpful in the prognosis of patients with advanced colorectal cancer. Therefore, there is an urgent need to find new targets for predicting and treating colorectal cancers. Recently, studies have found that the differentially expressed gene GNG4 plays an important role in tumor proliferation; however, GNG4 may have different mechanisms of action in different tumors. Studies by Yang et al. and Liang et al. [6, 9–12] have confirmed that GNG4 expression is significantly upregulated in colorectal cancer, which is related to prognosis [6], and may affect tumor progression through the PI3K-AKT signaling pathway (Figure 9). Palma et al. [5] has reported that GNG4 in primary advanced rectal cancer plays a critical role in the sensitivity of preoperative radiotherapy and chemotherapy. GNG4 expression is significantly upregulated in patients who are sensitive to radiotherapy and chemotherapy, which may be related to tumor cell growth and proliferation. Another similar study found that GNG4 was elevated in colorectal cancer patients who were sensitive to cetuximab treatment [13]. This shows that GNG4 may play a role in promoting tumor development in colorectal cancer, therefore representing a potential therapeutic target. However, other studies have found that GNG4 is hypermethylated in glioblastoma and bladder cancers, and its expression was significantly downregulated [3, 4]. Overexpression of GNG4 was also found to inhibit cell proliferation and colony formation [3]. Knockout of PSMC3IP in hepatocellular carcinoma cells has been shown to inhibit cell proliferation and colony formation, while TP53 and GNG4 genes were significantly upregulated [14].

To our knowledge, there has been no investigation on the function of GNG4 in colorectal cancer. In this study, we reported significantly increased expression of GNG4 in colorectal cancer. Colon cancer patients with high GNG4 expression had poor prognosis; however, the expression of GNG4 has no obvious relationship with prognosis in rectal cancer, which may be a result of fewer data points. *In vitro* studies have found that downregulation of GNG4 expression significantly inhibited cell proliferation, migration, and invasion and increased apoptosis and cell cycle arrest at the S phase.

Our research also has limitations. The prognostic sample size of colorectal cancer included is underpowered, which may affect the results. Although we conducted in vitro experiments with a colon cancer cell line, we lacked further verification by in vivo experiments. In the next step, we will verify it through more cell and in vivo experiments.

In conclusion, GNG4 may be an oncogene for colorectal cancer and may become a suitable new diagnostic marker and therapeutic target for colorectal cancer treatment.

## Figures and Tables

**Table 1:** Clinical characteristics of 55 cancer patients.

Characteristics	*N* (%)
*Gender*	
Female	21 (38.2)
Male	34 (61.8)

*Age (years)*	
≤55	22 (40)
>55	33 (60)

*TNM stage*	
I	13 (23.6)
II	11 (20)
III	17 (30.9)
IV	14 (25.5)

## Data Availability

The data used to support the findings of this study are available from the corresponding author upon request.
